# The Properties and Tortilla Making of Corn Flour from Enzymatic Wet-Milling

**DOI:** 10.3390/molecules24112137

**Published:** 2019-06-06

**Authors:** Jie Liu, Tiantian Yuan, Ruijuan Wang, Yawei Liu, Guihong Fang

**Affiliations:** 1 College of Food Science and Technology, Henan University of Technology, Zhengzhou 450001, China; liujie@haut.edu.cn (J.L.); yuantiantian0226@163.com (T.Y.); wrj0320@126.com (R.W.); 2 Department of Nutrition and Food Hygiene, Hainan Medical University, Haikou 570100, China

**Keywords:** enzymatic wet-milling, corn flour, tortillas, properties

## Abstract

Corn flour was prepared by wet-milling with the treatment of neutral protease and the gelatinization, thermal and rheological properties were analyzed. Tortilla was prepared with enzyme treated corn flour (ECF) and additives (xanthan gum and cassava starch) and the properties were analyzed. Compared with dry-milling corn flour (DCF) and wet-milling corn flour (WCF), the ECF had less average particle size (16.74 μm), higher peak viscosity and higher final viscosity of 2997 cP and 3300 cP, respectively. The thermal properties showed that ECF had higher ∆H and lower T_o_, T_p_ and T_c_. The G′ of ECF gel (6%, *w*/*w*) was higher than that of DCF gel and WCF gel. Dynamic viscoelastic measurement indicated that the tortillas made of ECF had lower G′ and G″ over the frequency range (0.1–100 rad/s) after adding xanthan gum and cassava starch. The gel structure of tortillas made of ECF was homogeneous in distribution of pores. The gelatinization, thermal and rheological properties of corn flour were improved by addition of neutral protease. The addition of xanthan gum and cassava starch helped to make the tortilla with porous structure and good sensory quality.

## 1. Introduction

Corn, as a staple food in Mexico and Central America, is one of the three great grain crops of the world with a global production of 1.045 billion tons in 2017/2018. Corn is used for production of an array of traditional and industrialized human food such as tortillas, corn chips, nachos, popped corn, porridges and corn grits [1]. Corn dry-milling by tempering-degerming is the method most commonly practiced by the industry and products are highly refined grits, meals, and flours. The corn wet-milling industries aim toward the extraction of the maximum possible amount of native or undamaged starch granules. Lime-cooking or nixtamalization of maize consists of cooking maize kernels in an alkaline (calcium hydroxide) solution, followed by stone grinding to produce masa, sheeting it, and forming it into thin discs and then baking into tortillas. The ancient nixtamalization process is presently adapted to high processing manufacturing procedures aimed toward the production of fresh and dry masa flours.

Traditional nixtamalization processes require about 12–16 h soaking time, 3 L of water and 1–2% of Ca(OH)_2_ per kilogram of corn [2]. Salazar [3] reported that the addition of Ca(OH)_2_ during nixtamalization could effectively reduce the content of acrylamide in tortilla chips. It is widely believed that the changes occurring in starch during nixtamalization are responsible for the textural and sensory properties of masa and its products [4,5]. Mondragón [6] reported that the viscoelastic behavior of nixtamalized starch gels was predominantly affected by the calcium interactions with starch. The ions promote cross-linking of the starch when lime contents go up to 0.2%. Currently, technology-based processes of nixtamalized have been explored, including the use of extrusion [7], ultrasound [8] and ohmic heating [2] to reduce contamination from the alkaline residues.

Enzymatic corn wet-milling is a process derived and different from conventional wet-milling. More starch granules were exposed by hydrolyzing proteins in the endosperm after grinding [9,10,11]. Enzymatic wet-milling was developed rapidly due to environmental friendliness. Johnston [12] used the protease to replace SO_2_ to release the starch granules from the corn endosperm. Ramírez [13] reported that the use of enzymes in a modified procedure for wet-milling made SO_2_ to reduce to levels sufficient to inhibit microbial activity and decreased the steeping time from 36 to 6 h. Alvoila [14] studied the effect of protease and transglutaminase on the microstructure and shelf stability of wheat dough and tortilla. The results showed that dough with the continuous matrix hydrolyzed into protein pieces, or cross-linked into thick protein strands and a nonhomogeneous continuous network, leading to shelf instability. 

In recent years, the texture and structure of foods are improved by combining cereal flour with hydrophilic colloid. Hydrocolloids, such as methylcellulose, xanthan gum, guar gum, Arabic gum are frequently applied in food as fat replacers, water binders, texturizers and adhesives [15,16]. Román-Brito [17] demonstrated that the addition of hydrocolloid to tortilla decreased the hardness and increased the flexibility and rubbery characteristics of tortillas. Xue [16] put the methylcellulose and xanthan gum into the batters formulated using of wheat, corn, and rice flour to research the rheological properties. They found that xanthan gum increased both G′_max_ and G″_max_, whereas methylcellulose increased G′_max_ but decreased G″_max_. A higher temperature and shorter time were required to gelatinize starch when the hydrocolloids were added to batter systems.

The purpose of this study is to develop a new method to produce masa flour and evaluate the masa flour by making tortillas. This method is different from lime-cooking or nixtamalization in procedures, commercial wet-milling in products, and dry-milling in properties of corn flour. Moreover, the aim to use the enzyme in this study is different from the isolation of starch or modification of starch reported in existing literature. Therefore, the enzymatic wet-milling corn flour was prepared and the physicochemical properties were analyzed. Enzymatic wet-milling corn flour and additives (xanthan gum and cassava starch) were used to make tortillas, and the analysis on the properties of tortillas was used to evaluate the procedures of enzymatic wet-milling.

## 2. Results and Discussion

### 2.1. Properties Analysis of Corn Flours

#### 2.1.1. Proximate Composition Analysis

Proximate composition varied significantly among three flours (Table 1). The starch content of wet-milling corn flour (WCF) (82.68%) and enzymatic wet-milling corn flour (ECF) (82.77%) was significantly (*p* < 0.05) higher than that of dry-milling corn flour (DCF) (75.04%), while the protein content of WCF (4.79%) and ECF (4.29%) was significantly (*p* < 0.05) lower than that of DCF (6.93%). It revealed that the enzymatic wet-milling and the wet-milling caused protein loss and more starch granules exposed [18]. The fat contents were significant different (*p* < 0.05) in three corn flours, which was highest for DCF (3.11%) and lowest for ECF (2.04%). The moisture content of DCF (11.22%) was significantly higher than that of WCF (7.85%) and ECF (8.05%). The variation in chemical composition between flours in the present study was due to different preparation methods. Both the content of protein and fat of three corn flours in this study were lower than that of the nixtamalization corn flour according to the report of Salazar [3].

#### 2.1.2. Morphological Properties 

**Figure 1:**
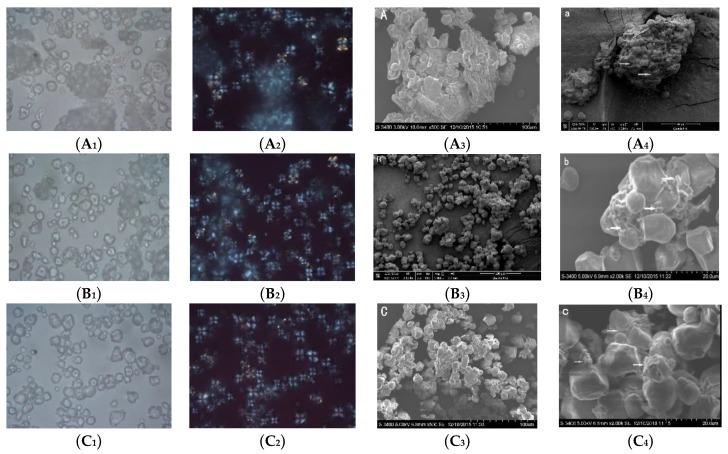
Morphological properties of corn flours. (**A**) dry-milling corn flour; (**B**) wet-milling corn flour; (**C**) enzymatic wet-milling corn flour. 1: Light microscopy of corn flour (10 × 50 times); 2: polarized light micrographs of corn flour (10 × 50 times); 3, 4: scanning electron microscopy of corn flour (with scale bars).

Polarized light micrographs and SEM image of corn flours are presented in Figure 1. More large particles was observed in DCF in Figure 1A, which might from corn endosperm fragments. The starch granules were embedded in the protein matrix (Figure 1A) and the exposed starch granules were less. The starch was partially released from protein matrix in WCF (Figure 1B). It might be due to water molecules can act as plasticizer diffused through the interstitial space, thus the protein matrix partially solubilized to release starch granules [7]. The starch granules of ECF were round to polygonal in shape which was consistent with Khatoon’s reports [19], and evenly dispersed (Figure 1C) compared with WCF and DCF. The starch granules were embedded in protein matrix to form continuous phase which might be corn endosperm (Figure 1A_4_, ×2000). However, smooth starch granule surface and small amount of protein adherence could be observed in Figure 1C_4_ (×2000). It revealed that the enzymatic wet-milling was an effective method using neutral protease to release the starch granules from the corn endosperm. This result was consistent with previous reports [11].

#### 2.1.3. Granule Size 

The mean granule size of DCF, WCF and ECF were 43.67 µm, 18.92 µm, 16.74 µm, respectively. There were extremely significant differences (*p* < 0.01) in the granule size distribution of the three corn flours (Table 1). This result was consistent with the SEM image. It indicated that high protein content of the corn flour led to large granule size [20]. The mean granule size of ECF was comparable to that of corn starch (16.58 µm) from laboratory corn wet-milling reported by Wang [21]. It indicated that the hydrolysis of protein by neutral protease effectively reduced the granule size of corn flour.

#### 2.1.4. Crystalline Texture

The three corn flours showed typical A type diffraction pattern with peaks at 15.3°, 17.1°, 23.5° (2θ) (data not shown). The crystallinity of ECF, WCF and DCF were 32.43%, 30.40%, 27.18%, respectively (Table 1). In general, the crystallinity was related to starch in corn flours. Crystallinity decreased with the increase of the damaged starch granules by milling [22,23]. The starch granules were damaged during dry-milling, resulting in a decrease in crystallinity [24]. Wet-milling is milling of corn in excess water. Water molecules could increase the elasticity of starch granules, thereby increasing the fracture toughness of the starch granules and minimizing the damage to the starch granules [25]. Enzymatic wet-milling adding neutral protease diminished the combination of starch and protein, and lowered starch damage in subsequent milling.

#### 2.1.5. Pasting Properties 

**Figure 2:**
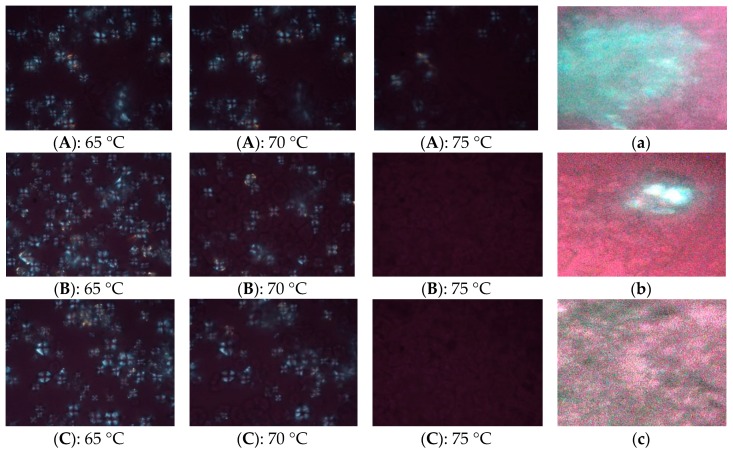
Polarized light micrographs of corn flour heated in the different temperature. (**A**,**a**): dry-milling corn flour; (**B**,**b**): wet-milling corn flour; (**C**,**c**): enzymatic wet-milling corn flour. (**A**–**C**): polarized light image of corn flour gelatinized at 65 °C, 70 °C, 75 °C (10 × 50 times). (**a**–**c**): DSC-scanned corn flour at a microscope (10 × 50 times).

Gelatinization is a phase transition of starch granules from an ordered state to a disordered state during heating with excess water. Disordered states include melting of ordered regions at the level of crystallite (inner and surface) and amylopectin double-helical order [26]. Figure 2 shows the polarized light micrographs of corn flour gelatinized at 65 °C, 70 °C, 75 °C. Compared with 65 °C, it was obvious that the maltese cross disappeared more than half in WCF and ECF at 70 °C. At 75 °C, there was a small amount of maltese cross in the DCF, while the maltese cross of WCF and ECF completely disappeared. 

The gelatinization characteristic values of corn flour were summarized in Table 2. The P_temp_ of the three corn flours varied from 78.23 to 80.45 °C. The P_temp_ of DCF was the highest, indicating that starch granules in DCF were not easy to swell and rupture [27]. The PV, TV, FV and SV of ECF were higher than that of DCF and WCF. During the cooking stage (temperature from 50 °C to 95 °C), the increase in viscosity of the corn flour paste demonstrated the water absorption and swelling ability of the starch [28]. During the cooling stage (temperature from 95 °C to 50 °C), the increase in viscosity of the system was attributed to the aggregation of amylose itself by hydrogen bonds [29]. It indicated that the protein associated with starch granules restricted swelling and amylose leaching, and reduced the viscosity of the corn flour paste during the gelatinization process. The results were in accordance with the previous reports [30,31,32,33,34].

There were significant differences in T_o_, T_p_, T_c_ and ∆H of corn flour (*p* < 0.05). The T_o_ (69.99 °C), T_p_ (75.36 °C) and T_c_ (79.32 °C) of DCF was higher than that of WCF and ECF. The difference in gelatinization temperature between corn flour may be attributed to the granule size of starch and distribution of starch [27]. ∆H, reflecting the energy absorption of corn flour gelatinization, was influenced by the morphology, size and distribution of starch granules [26]. The ∆H of the three corn flours ranged from 6.59 to 13.43 J/g (Table 3). The ∆H (13.43 J/g) of the ECF was the maximum, and the ∆H of the DCF (6.59 J/g) was minimum (*p* < 0.05). This result could be explained by degree of crystallinity. The crystallinity of ECF, WCF and DCF was 32.43%, 30.40% and 27.18%, respectively. 

The samples were recycled after DSC-scanned and observed under a light microscope. There were aggregation structure in DCF and WCF (Figure 2a,b). The aggregation structure of DCF was larger than that of WCF (Figure 2a,b). ECF formed homogeneous paste after DSC-scanned. This result indicated that some starch granules were trapped in the protein matrix in WCF and DCF and were not gelatinized, while starch granules in ECF could be gelatinized completely. 

#### 2.1.6. Rheological Properties

During the gelatinization process, the starch granules in the corn flour swelled, accompanied by the dissolution of the particulate components (mainly amylose) and the formation of a three-dimensional network structure. The rheological properties of corn flour depend on the shape of the particles, the particle size distribution, the interaction between the particles and the viscosity of the continuous phase [33]. The paste and gel after gelatinization were analyzed.

**Figure 3:**
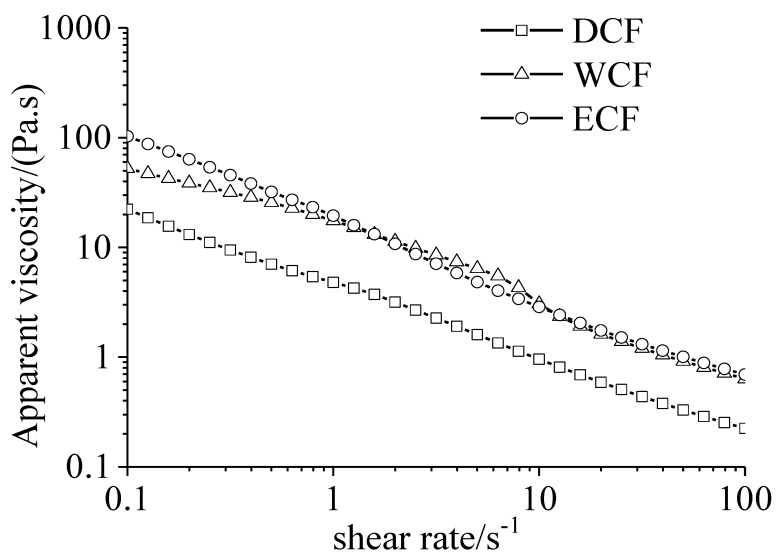
Behavior of the apparent viscosity of corn flour. DCF: dry-milling corn flour; WCF: wet-milling corn flour; ECF: enzymatic wet-milling corn flour.

The apparent viscosity of three corn flour pastes decreased with the increase of shear rate from 0.1 to 100 s^−1^ (Figure 3), showing the properties of non-Newtonian fluid (*n* < 1). The relationship between shear stress and shear rate of the three corn pastes was fitted by the Herrschel-Bulkey model, and the parameters were summarized in Table 4. The equations had a good correlation with the curves (R^2^ = 0.97–0.99). The apparent viscosity of the ECF and WCF paste was higher than that of the DCF paste (Figure 3). The difference of apparent viscosity may be due to the difference of protein content in corn flour. The existence of protein in corn flour restricted the hydration and swelling of starch granules, resulting in incomplete gelatinization [33]. 

**Figure 4:**
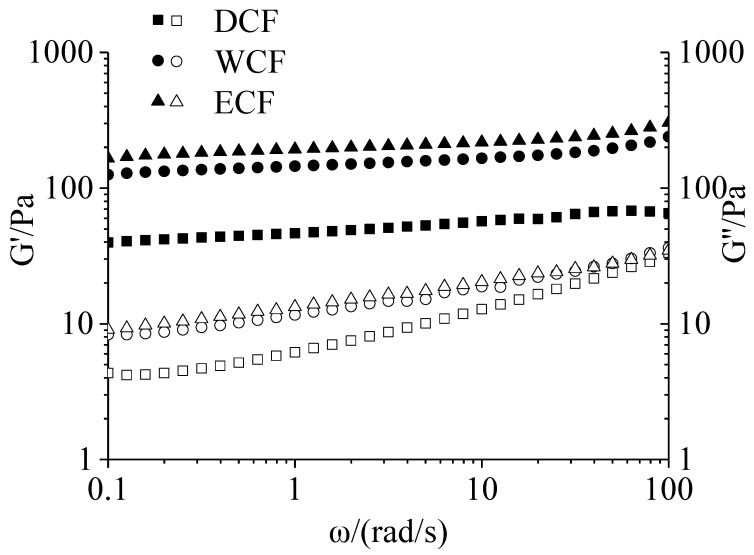
Frequency sweep of corn flour gel. DCF: dry-milling corn flour; WCF: wet-milling corn flour; ECF: enzymatic wet-milling corn flour. Storage modulus G′ (■, ▲, ●), loss modulus G″ (□, ∆, ○).

The frequency sweep of corn flour gels is shown in Figure 4. G′, a measure of deformation energy stored in the samples during shearing, represents the elastic behavior of the samples, while G″, a measure of deformation energy used up and lost during shear, represents the viscous behavior of the samples. The G′ was higher than G″ of the three samples (Figure 4) at a frequency of 0.1–100 rad/s. This result indicated that the elasticity of the samples played a major role in the gel structure [35]. Over the frequency range of 0.1–100 rad/s, the G′ of the three samples did not change significantly, and G″ increased slightly when the frequency increased. It revealed that a typical biopolymer gel network structure was formed in corn flour. The G′ of ECF and WCF was higher than that of DCF. It indicated that the ECF and WCF had higher degree of resistance to deformation than DCF. For the G″, the ECF and WCF was high than DCF. This may be due to the fact that the protein content of ECF and WCF was lower than DCF, which weakened the restriction on starch and made starch to flow easily. 

### 2.2. Properties Analysis of Tortillas 

#### 2.2.1. Micro-structure Analysis

**Figure 5:**
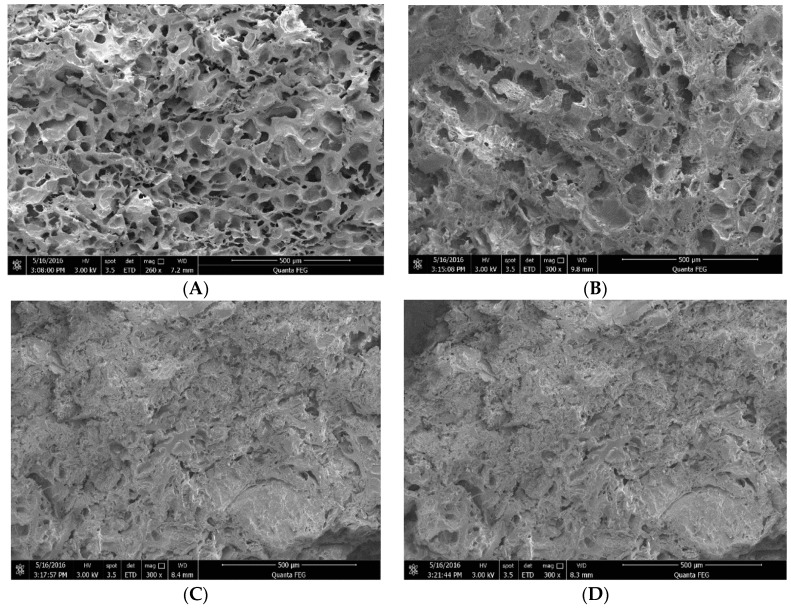
SEM micrographs of tortillas (with scale bars). (**A**): enzymatic wet-milling corn flour + 0.5% xanthan gum + 5% cassava starch; (**B**): enzymatic wet-milling corn flour; (**C**): dry-milling corn flour + 0.5% xanthan gum + 5% cassava starch; (**D**): dry-milling corn flour.

The cross section structure of the tortillas was observed by SEM (Figure 5). The tortilla prepared by ECF had many evenly distributing pores (Figure 5B), while the tortilla prepared by DCF had less pores (Figure 5D). Xie [36] reported that the lower viscosity of the paste was not favorable to form pores in uniform size. After the addition of xanthan gum and cassava starch, the tortilla prepared by ECF possessed a porous structure of homogeneous distribution (Figure 5A). Renzetti [37] reported that the hydrocolloids had the function of confer elasticity and gas holding of foods, while for the DCF, the addition of xanthan gum and cassava starch did not affect the gel structure of tortillas (Figure 5C). The difference in gel structure of tortillas was related to the gelatinization of corn flour in the formula flour, the dissolution of amylose and the aggregation of protein and xanthan gum [38].

#### 2.2.2. Dynamic Rheological Properties of the Tortillas 

**Figure 6:**
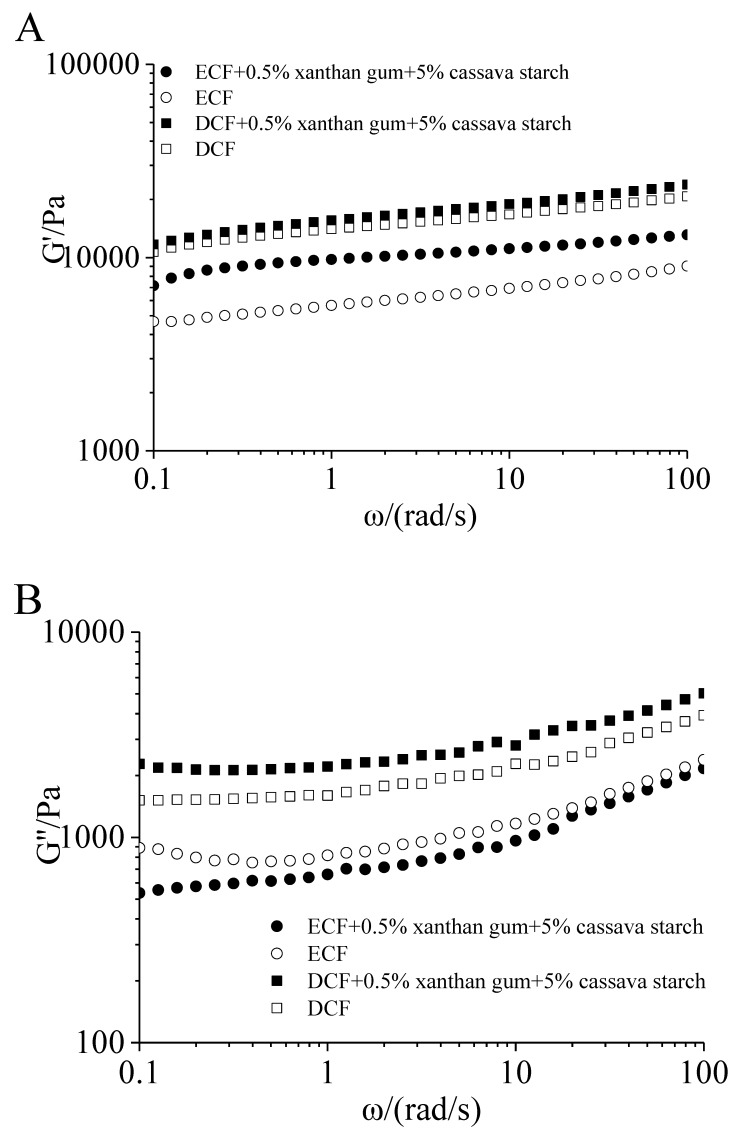
Frequency sweep of tortillas. (**A**): Storage modulus G′; (**B**): loss modulus G″. DCF: dry-milling corn flour; WCF: wet-milling corn flour; ECF: enzymatic wet-milling corn flour. ●: enzymatic wet-milling corn flour + 0.5% xanthan gum + 5% cassava starch; ○: enzymatic wet-milling corn flour; ■: dry-milling corn flour + 0.5% xanthan gum + 5% cassava starch; □: dry-milling corn flour.

The frequency sweep of the four tortillas is shown in Figure 6. The G′ of the four samples was higher than G″ at the frequency range of 0.1–100 rad/s, which indicated that the tortillas possessed the properties of solid elasticity. The G′ of tortillas prepared by DCF was higher than that of ECF (Figure 6A). The G′ of tortillas increased after the addition of 0.5% xanthan gum and 5% cassava starch, indicating that the addition of xanthan gum strengthened the gel structure of the tortillas. It may be ascribed to form the network structure of copolymer through the synergistic interaction between corn flour and xanthan gum. The result was consistent with the study of Soulef [39]. The G″ of tortillas prepared by DCF were higher than that of ECF. The addition of additives decreased the G″ of tortilla prepared by ECF, while increased in that of tortilla prepared by DCF. The additives have different effects on the G″ of ECF and DCF tortillas, which may be related to the addition of cassava starch [39]. 

#### 2.2.3. Hardness of Tortillas

**Figure 7:**
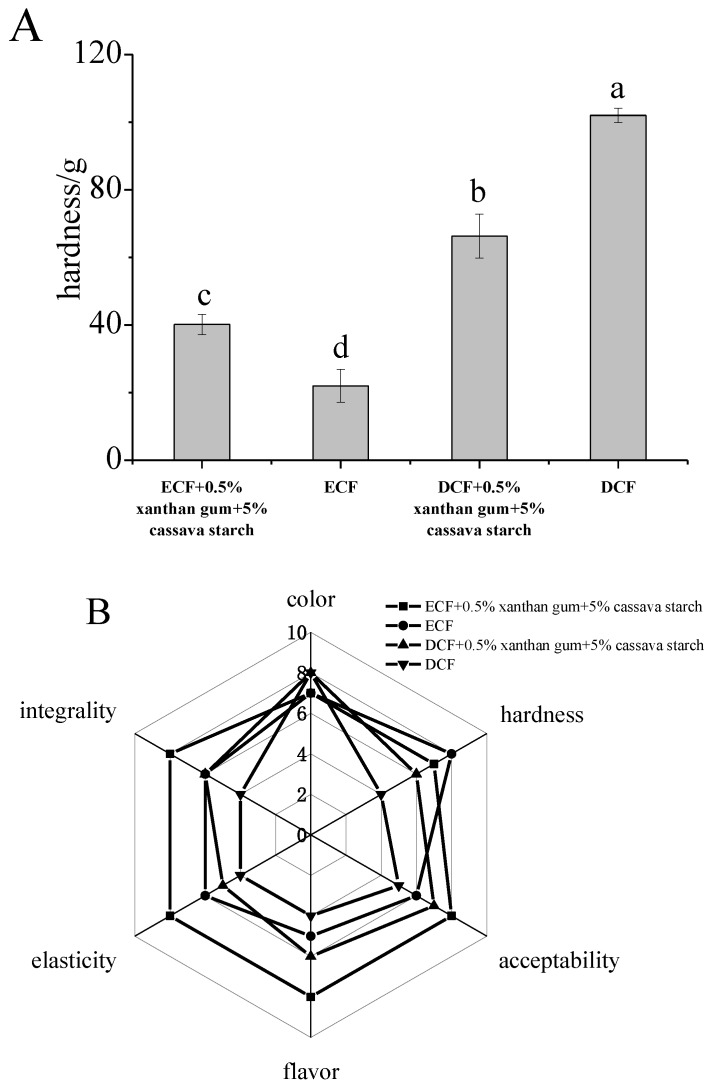
The hardness and sensory evaluation of tortillas. (**A**): the hardness of tortillas; (**B**): sensory evaluation of tortillas. DCF: dry-milling corn flour; WCF: wet-milling corn flour; ECF: enzymatic wet-milling corn flour. Different letters in (A) indicate values are significantly different (*p* < 0.05). ■: enzymatic wet-milling corn flour + 0.5% xanthan gum + 5% cassava starch; ●: enzymatic wet-milling corn flour; ▲: dry-milling corn flour + 0.5% xanthan gum + 5% cassava starch; ▼: dry-milling corn flour.

There was a significant difference (*p* < 0.05) between the hardness of the four tortillas (Figure 7A). The hardness of tortilla prepared by DCF was the highest, while it was lowest in tortilla prepared by ECF. The hardness of tortilla prepared by DCF was significant decreased (*p* < 0.05) after the addition of xanthan gum and cassava starch. Luis [35] illustrated that a soft texture and pleasant flavor could be obtained by the addition of cassava starch to tortillas. Therefore, the overall hardness of DCF was reduced after the addition of cassava starch replacing part of DCF. The hardness of tortilla prepared by ECF was significant increased (*p* < 0.05) after the addition of xanthan gum and cassava starch, which was consistent with some previous studies [40,41,42]. Achayuthakan [43] reported that the addition of xanthan gum promoted the formation of a three-dimensional network and increased the hardness of tortillas. The difference in hardness of tortillas prepared by DCF and ECF after the addition of xanthan gum and cassava starch could observed by SEM image. The tortilla made by ECF with xanthan gum and cassava starch had moderate hardness and porous structure. The particle size of corn flour and the combination of starch and protein in the granules could affect the gelatinization of corn flour and protein aggregation during the preparation of tortillas, further affect the structure of the tortilla [44].

#### 2.2.4. Sensory Evaluation of Tortillas

The flavor, integrality and acceptability of tortillas were improved with adding xanthan gum and cassava starch (Figure 7B). The tortillas obtained the highest sensory score was made by ECF with 0.5% xanthan gum and 5% cassava starch, and it was refreshed, non-sticky and not obvious graininess. In the case of hardness, the results were consistent with the previous analysis (Section 2.2.3). The slight difference in color among samples may be due to the different protein content. Rendón-Villalobos [45] prepared tortillas with fiber-rich by replacing part of corn flour with chía seed and obtained good sensory evaluation. The addition of xanthan gum had a similar effect.

## 3. Materials and Methods 

### 3.1. Materials

Corn (Xiannong Seed Co., LTD, Nanchang, China); the content of starch, protein and fat was 71.52%, 8.50% and 3.88%, respectively; the bulk density and relative density was 778.71% and 1.29%, respectively) and cassava starch (Hengrui Technology, Luohe, China) were obtained from a local supermarket. Neutral protease (1.27 × 105 U/mL) was obtained from Novozymes (Copenhagen, Denmark). Xanthan gum and guar gum was obtained from Danisco (Wilmington, DE, USA).

### 3.2. Preparation

#### 3.2.1. Corn Flour Preparation

Corn flour was prepared from enzymatic wet-milling by the following procedures: 50 g of corn was soaking in 150 mL of water in a thermostat water bath (Itherm-B3 vivo, VIVO Technology Co., Ltd., Seelbach, Germany) at 50 °C for 6 h and coarsely ground in a knife blender (Guohua Instrument Co., Ltd., Changzhou, China) for 1 min. Then the slurry was placed in a water bath at 50 °C and adjusted to pH 6.6. Neutral protease (0.35 mL) was mixed into the slurry for enzyme treatments for 7 h. The sample was ground in a colloid mill (JMS-30A; Langfang Langtong Machinery Co., Ltd., Langfang, China) at rotor speed of 4500 r/min after enzyme inactivation and passed through a 100 mesh sieve. The wet cake of corn flour was obtained by vacuum filtration (SHZ-D III; Gongyi Yuhua Instrument Co., Ltd., Zhengzhou, China). The corn flour were dried in an oven at 40 °C for 12 h after washing 3 times with 500 mL deionized water.

Wet-milling experiments were done followed the conditions described above without the addition of enzyme.

Dry-milling process was done by a cyclone mill (FS-II; Zhejiang Top Instrument Co., Ltd., Hangzhou, China) and the flour were passed through a 100 mesh sieve.

#### 3.2.2. Tortilla Preparation

Xanthan gum, guar gum and cassava starch mixed with ECF were used for the orthogonal experiment (data not shown). The optimum additives (xanthan gum, 0.5%, g/100 g; cassava starch, 5%, g/100 g) obtained by orthogonal experiments were applied to prepare tortilla. The formula flour was hydrated with distilled water (formula flour: water was 1:3) and blended manually for 2 min, and then the mixture was molded into thin circles (30 g). The thickness and the diameter of the corn dough was about 3 mm and 80 mm, respectively. The corn dough were baked at 200 °C for 6 min and packed into polyethylene bags after cooling to avoid moisture loss and stored at room temperature for evaluation.

### 3.3. Properties Analysis of Corn Flour

#### 3.3.1. Proximate Analysis

Determination of starch content in corn flour by 1%-hydrochloric acid optical rotation method. Protein content of the corn flour was measured using AACC Approved method 46-13A (AACC International, 2000, Method 44-13A). Moisture and fat content were determined according to Approved method 44-15.02 (AACC International, 1995, Method 44-15.02) and GB/T 5512-2008, respectively.

#### 3.3.2. Scanning Electron Microscopy (SEM)

Corn flour was first sprinkled on double-sides adhesive tape, which was attached to aluminum stubs, and the sample were then plated with a thin layer of gold. By using a high vacuum bench top scanning electron microscope (Quanta250 FEG; FEI, Hillsboro, OR, USA), the sample were observed and photographed.

#### 3.3.3. Particle Size 

The size distribution of corn flour was determined by laser diffraction particle size analyzer (SALD-301V; SHIMADZU, Kyoto, Japan). Prior to analysis, the samples were dispersed in distilled water to form a suspension (2%, *w*/*v*).

#### 3.3.4. Light Microscopy (LM)

The morphology and birefringence of corn flour were observed in a digital camera belonged to the optical microscope (ECLIPSE 50i POL; Nikon, Kanagawa, Japan). Corn flour suspension (2%, *w*/*v*) were placed on the microscope slide with a glass coverslip. Then the samples was observed under the polarized light microscope.

#### 3.3.5. X-ray Diffraction (XRD)

Crystalline properties were assessed using an X-ray diffractometer (Rigaku MiniFlex600, Tokyo, Japan), which was operated at 30 mA and 40 KV. The diffraction angle (2θ) scanned was from 5° to 45° with a scanning speed of 4°/min. The crystallinity of corn flour was obtained by integrating the diffraction pattern with software MDI JADE 6.0.

#### 3.3.6. Thermal Properties

The thermal properties of the corn flour were determined using a differential scanning calorimeter (DSC) (Q20; TA Instruments, New Castle, DE, USA), and nitrogen purge gas was used in the experimental work. Corn flour samples (3.0 mg, db) were wetted with distilled water (7 μL) in an aluminum pan and immediately sealed. The sealed pans were allowed to stand for 24 h at room temperature before the measurements. The sample pans were scanned from 30 to 100 °C with a heating rate of 10 °C/min and an empty pan was used as a reference [27]. The DSC equipment software was applied to analyze onset temperatures (To), peak temperatures (Tp), final temperatures (Tc) and enthalpy gelatinization enthalpy (∆H).

#### 3.3.7. Pasting Properties

Pasting properties of corn flour (8.0% db, 28 g total weight) were determined using a Rapid Visco-Analyser (RVA) (Perten Instruments Ltd.; Sydney, Australia). The heating and cooling cycles were programmed in the following manner. The slurry was held at 50 °C for 30 s, heated to 95 °C within 2.5 min and then held for 20 min. It was subsequently cooled to 50 °C within 3 min and then held for 9 min [46]. Peak viscosity (PV), trough viscosity (TV), final viscosity (FV), breakdown (BD) and setback viscosity (SV) were evaluated.

#### 3.3.8. Rheological Properties

Rheological experiments were carried out using a DHR-1 rheometer (TA Instruments, New Castle, DE, USA). A 40 mm diameter parallel plate was used and the gap between the plates was set to 1 mm. The free surface of the sample edges was covered with silicone oil to prevent evaporation of the sample during the measurements [47]. The temperature was controlled by a water bath connected to the peltier system.

##### Steady Shear

Flour suspensions (5%, db) was prepared in deionized water and gelatinized by a Rapid Visco-Analyser in order to obtain starch paste and the experimented method was described in Section 3.3.7. The starch pastes was loaded on a peltier-plate carefully. The steady shear tests were performed at 25 °C over the shear rate range of 0.1–100 s^−1^ and apparent viscosity was recorded as a function of shear rate. The relationship of shear stress and shear rate were fitted to the Herschel-Bulkley model [48].

(1) $$\tau \;=\;\tau_{0}+K\gamma^{n}$$

where *τ* is shear stress (Pa), *τ*_0_ is yield stress (Pa), *K* is the consistency coefficient (Pa·s^n^), *γ* is shear rate (1/s), and *n* is the flow behavior index (dimensionless).

##### Frequency Sweep

For frequency sweep experiments, the samples (6%, db) were prepared according to the method depicted in Section 3.3.7. The starch pastes were then standing 1 h at room temperature before the test. A resting time of 5 min was allowed before the frequency sweep was performed from 0.1 to 100 rad/s at 1% strain (within the linear viscoelastic region) at 25 °C. The viscoelastic parameters obtained with frequency were the storage modulus (G′), loss modulus (G″).

### 3.4. Properties Analysis of Tortilla

#### 3.4.1. Scanning Electron Microscopy (SEM)

The tortillas were dried in a freeze dryer for 24 h to observe the micro-structure of the cross section. The method was showed in Section 3.3.2.

#### 3.4.2. Hardness of Tortillas

The hardness of the tortilla was determined by puncture test (TA.XT.Plus Texture Analyser; Stable Micro System, Godalming, UK). A probe (HDP/TPB) at a constant rate of 0.5 mm/s and the compressive stress of 30% was used. Maximum breaking force was the hardness of the tortilla [49].

#### 3.4.3. Dynamic Rheological Properties (Frequency Sweep)

A piece of tortilla with 40 mm diameter was cut from the central part of tortilla for measurement. The frequency sweep was examined at 25 °C from 0.1 to 100 rad/s at 0.5% strain (within the linear viscoelastic region) [50]. The viscoelastic parameters obtained with frequency were the storage moduli (G′), and loss moduli (G″).

#### 3.4.4. Sensory Evaluation of Tortillas

A panel of 12 trained persons carried out the tortilla sensory evaluation. The panel evaluated five attributes (hardness, elasticity, color, integrality and overall sensory acceptance) using a hedonic scale, where number 10 to 1 corresponded to the decrease in the level of preference [51].

### 3.5. Statistical Analysis

All measurements were done in triplicate. Data in all the tables were reported as mean ± standard deviation (SD, *n* = 4). Duncan’s Pairwise Comparisons was used to analyze the difference of mean values, and *p* < 0.05 was considered to be statistically significant during operating the analysis of variance (ANOVA) test.

## 4. Conclusions

ECF was prepared and the characteristics were analyzed based on laboratory wet processing combined with enzyme treatment technology. The preparation of ECF and the determination of the characteristics of three corn flours (ECF, WCF, DCF) revealed that neutral protease could effectively hydrolyze protein in corn endosperm and weaken the combination of starch and protein, improving the gelatinization, thermal and rheological properties of corn flour. The prepared tortilla possessed porous structure and good sensory quality after the addition of xanthan gum and cassava starch. Therefore, application of neutral protease could be favorable to corn enzymatic processing and the quality of tortilla could be significantly improved by a combination of hydrophilic colloid and ECF.

## Figures and Tables

**Table 1:** The chemical composition analysis, size distribution and crystallinity of corn flours.

Parameters	DCF	WCF	ECF
Starch content (%)	75.04 ± 0.75 ^b^	82.68 ± 0.67 ^a^	82.77 ± 0.06 ^a^
Protein content (%)	6.93 ± 0.01 ^a^	4.79 ± 0.01 ^b^	4.29 ± 0.07 ^b^
Fat content (%)	3.11 ± 0.01 ^a^	2.59 ± 0.01 ^b^	2.04 ± 0.01 ^c^
Moisture content (%)	11.22 ± 0.07 ^a^	7.85 ± 0.03 ^c^	8.05 ± 0.10 ^b^
Other (%)	3.70 ± 0.05 ^a^	2.09 ± 0.06 ^c^	2.85 ± 0.10 ^b^
Mean granule size (μm)	43.68 ± 0.67 ^a^	18.92 ± 0.10 ^b^	16.74 ± 0.07 ^c^
d (10%) (μm)	16.51 ± 0.46 ^a^	13.97 ± 0.11 ^b^	11.74 ± 0.11 ^c^
d (50%) (μm)	37.91 ± 0.74 ^a^	18.41 ± 0.11 ^b^	16.74 ± 0.11 ^c^
d (90%) (μm)	141.71 ± 0.42 ^a^	26.56 ± 1.08 ^b^	24.43 ± 0.41 ^c^
Crystallinity (%)	27.18 ^c^	30.40 ^b^	32.43 ^a^

DCF: dry-milling corn flour; WCF: wet-milling corn flour; ECF: enzymatic wet-milling corn flour. Different letters (^a–c^) within the same row indicate values are significantly different (*p* < 0.05). d (10%): 10% of the corn flour granules smaller than this value; d (50%): 50% of the corn flour granules smaller than this value; d (90%): 90% of the corn flour granules smaller than this value.

**Table 2:** Pasting properties of corn flour.

Samples	PV (cP)	TV (cP)	BV (cP)	FV (cP)	SV (cP)	P_temp_ (°C)
DCF	1328 ± 4 ^c^	626 ± 6 ^c^	702 ± 2 ^b^	2715 ± 28 ^b^	2089 ± 22 ^a^	80.45 ± 0.40 ^a^
WCF	2964 ± 3 ^b^	1099 ± 23 ^b^	1866 ± 26 ^a^	3157 ± 48 ^a^	2058 ± 25 ^a^	78.23 ± 0.03 ^b^
ECF	2997 ± 4 ^a^	1204 ± 12 ^a^	1793 ± 16 ^a^	3300 ± 12 ^a^	2096 ± 24 ^a^	79.45 ± 0.15 ^a^

DCF: dry-milling corn flour; WCF: wet-milling corn flour; ECF: enzymatic wet-milling corn flour. Different letters (^a–c^) within the same column indicate values are significantly different (*p* < 0.05).

**Table 3:** Thermal properties of corn flour.

Samples	T_o_ (°C)	T_p_ (°C)	T_c_ (°C)	∆H (J/g)
DCF	69.99 ± 0.17 ^a^	75.36 ± 0.01 ^a^	79.32 ± 0.47 ^a^	6.59 ± 0.14 ^c^
WCF	68.36 ± 0.07 ^b^	72.75 ± 0.05 ^b^	78.33 ± 0.04 ^a^	12.63 ± 0.02 ^b^
ECF	68.54 ± 0.31 ^b^	72.89 ± 0.09 ^b^	78.41 ± 0.25 ^a^	13.43 ± 0.00 ^a^

DCF: dry-milling corn flour; WCF: wet-milling corn flour; ECF: enzymatic wet-milling corn flour. Different letters (^a–c^) within the same column indicate values are significantly different (*p* < 0.05).

**Table 4:** The Hereschel-Bulkey model parameters of corn flour paste.

Samples	*τ*_0_ (Pa)	K (Pa·s)	n	R^2^
DCF paste	2.4254 ± 0.1830 ^c^	2.0536 ± 0.2307 ^b^	0.5019 ± 0.0195 ^b^	0.9944 ± 0.0008 ^a^
WCF paste	11.9076 ± 0.4290 ^b^	3.6578 ± 0.4430 ^a^	0.5793 ± 0.0053 ^a^	0.9797 ± 0.0117 ^a^
ECF paste	13.0624 ± 0.0024 ^a^	4.0435 ± 0.0199 ^a^	0.5766 ± 0.0038 ^a^	0.9942 ± 0.0039 ^a^

DCF: dry-milling corn flour; WCF: wet-milling corn flour; ECF: enzymatic wet-milling corn flour. Different letters (^a–c^) within the same column indicate values are significantly different (*p* < 0.05).
